# Corrosion Behavior of the CoNiCrAlY-Al_2_O_3_ Composite Coating Based on Core-Shell Structured Powder Design

**DOI:** 10.3390/ma14227093

**Published:** 2021-11-22

**Authors:** Wenmin Guo, Honglin Zhang, Shan Zhao, Zhibing Ding, Bin Liu, Wenjuan Li, Huanhuan Xu, Huiyuan Liu

**Affiliations:** 1 State Key Lab of Powder Metallurgy, Central South University, Changsha 410083, China; wenminguo@hotmail.com; 2 College of Mechanical and Energy Engineering, Shaoyang University, Shaoyang 422000, China; zhanghonglin202011@yeah.net (H.Z.); aigsrgsy510105@163.com (S.Z.); dzbing1992@163.com (Z.D.); lwjdone@163.com (W.L.); xh18183431279@163.com (H.X.); 3 Key Laboratory of Hunan Province for Efficient Power System and Intelligent Manufacturing, Shaoyang University, Shaoyang 422000, China; 4 Falcontech Co., Ltd., Wuxi 214145, China; huiyuan.liu@falcontech.com.cn

**Keywords:** core-shell structured powder, high velocity oxy-fuel spray, microstructure, corrosion

## Abstract

The oxidation of the metal powder during the thermal spraying process usually leads to significant deterioration of the microstructure and performance of the coating. In order to isolate the metal powder from oxygen during the spraying process, the CoNiCrAlY-Al_2_O_3_ core-shell structured powder with Al_2_O_3_ as the shell was designed in this study. The influence of the core-shell structured powder on the microstructure and corrosion resistance of the HVOF coating has been studied in detail. The results show that the temperature field of the molten CoNiCrAlY powder during the spraying process is significantly changed by the Al_2_O_3_ shell. The poor deformability of the CoNiCrAlY-Al_2_O_3_ droplets leads to an increase in the porosity and unmelted particles of the coating. In addition, the significant difference is that the coating also maintains a high content of β-NiAl phase. The lower oxide content in the CoNiCrAlY-Al_2_O_3_ coating indicates that the core-shell structured powder significantly inhibits the oxidation of the CoNiCrAlY core powder during the spraying process. The CoNiCrAlY-Al_2_O_3_ coating exhibits high corrosion potential, passive film resistance, charge transfer resistance, and low corrosion current density in 3.5 wt.% NaCl solution, indicating that the coating has excellent corrosion resistance.

## 1. Introduction

Under the comprehensive attack of corrosion, work load, and environmental load (mainly from wind, waves, and currents), the moving parts of marine engineering equipment suffered from complex service conditions where corrosion and wear coexist, such as deep-sea exploration and mining equipment, marine hydraulic transmission, underwater operation machinery and equipment, etc. [1,2]. Metal structural materials in geothermal steam power plants were often exposed to high concentrations of chloride and hydrogen ions, and are also highly susceptible to corrosion [3]. In view of the corrosion problem of metal materials, scholars have explored a series of effective corrosion protection methods, such as the corrosion inhibitors, electrochemical protection, surface coating technologies, and so on. Thermal spray coating technology is one of the effective methods for corrosion protection of metal structural materials. However, under normal circumstances, metal powder is unavoidably oxidized during the thermal spraying process. Then, multiple oxide layers are formed inside the coating, resulting in a typical layered structure of the thermal sprayed coating. A large number of studies have revealed that the oxide layer leads to significant deterioration of the coating performance. Deshpande et al. [4] found that the oxide film significantly reduced the wettability between droplets, resulting in a large number of mechanical bonding inside the coating, and ultimately reducing the bonding strength of the coating. Choi et al. [5] indicated that the oxidation of droplets can also cause changes in the local chemical composition of the material and initiate phase changes. In addition, the internal oxide layer of the coating usually leads to cracks, which reduce the fracture toughness [6,7]. The multiple interfaces of the layered structure caused by oxides usually become preferential channels for corrosion, decreasing their service life [8].

Regarding the oxidation of droplets during thermal spraying, scholars have conducted a lot of research on vacuum thermal spraying, nitrogen or argon atmosphere protection spraying, and cold spraying technologies. E. Vetrivendan et al. [9] used argon shielded plasma spraying (ASPS) technology to prepare a tantalum coating on the Cp-Ti substrate. The coating can be stored for up to 240 h in 11.5 M HNO_3_ + 0.05 M NaF solution, showing excellent corrosion resistance. However, the use of inert gas protection will result in higher coating preparation costs. Furthermore, the thermal spray system will become complicated, and the size of the workpiece will also be limited [10].

In recent years, a powder material with a core-shell structure has received extensive attention from researchers [11,12,13,14]. The core-shell structured composite powder is composed of core material and shell material. This special structure can organically combine the special properties of different substances and make up for the shortcomings of a single structure [15]. In order to prevent the core material from being oxidized, the shell material can be selected from high melting point or chemically stable inorganic oxides (Al_2_O_3_, SiO_2_, etc.). In addition, these high-hardness oxides can also improve the oxidation resistance of the coating and serve as a reinforcing phase inside the coating to improve its mechanical properties. Therefore, a reasonable core-shell structured powder design has become a key factor in the control of the microstructure and performance of the coating. Jiang et al. [16] reported a core-shell structured powder with Ni/Al as the outer shell and iron-based amorphous powder as the core material, which was prepared by mechanical ball milling. Then, the coating was prepared using a plasma spraying process. The results show that the corrosion resistance of the composite coating in 3.5 wt.% NaCl solution is significantly better than that of Fe-based amorphous coating. Tian et al. [17] prepared a NiCr-20Mo coating with core-shell structured powder. The results show that the oxide content of NiCr-20Mo coating is only 0.32%, which is significantly lower than 0.92% of NiCr coating. This shows that the core-shell structured powder effectively inhibits the oxidation of the core material during the thermal spraying process [18]. In addition, the corrosion current density of NiCr-20Mo coating in 3.5 wt.% NaCl solution is only 0.2 times that of NiCr coating, which shows excellent corrosion resistance.

Literature studies have shown that the integrity of the core-shell structure and the sphericity of the powder are critical to the microstructure and performance of thermal spray coatings [19]. In recent years, MCrAlY (M = Ni, Co or NiCo) alloy has been widely studied as a common coating material [20,21]. The γ and β phases that make up the CoNiCrAlY coating have similar self-corrosion potentials and are not prone to galvanic corrosion [22]. The appropriate amount of Cr element in the coating is conducive to the formation of a passivation film rich in Cr_2_O_3_ in a corrosive environment and further enhances the corrosion resistance of the coating [23]. In addition, Al_2_O_3_ can effectively inhibit the oxidation of CoNiCrAlY particles during thermal spraying and can improve the corrosion resistance of CoNiCrAlY coatings [24]. In our previous work, the preparation process of CoNiCrAlY-Al_2_O_3_ core-shell structured powder has been systematically optimized [25]. However, there is no research report on the influence of core-shell structured powder on the microstructure and corrosion resistance mechanism of CoNiCrAlY-Al_2_O_3_ composite coating. Therefore, in this work, we selected CoNiCrAlY and CoNiCrAlY-Al_2_O_3_ core-shell structured powder as raw materials, and prepared coatings on the surface of 304 stainless steel by high velocity oxy-fuel spray (HVOF) technology. The effect of the core-shell structured powder on the microstructure of the coating was studied. The corrosion resistance mechanism of these coatings in 3.5 wt.% NaCl solution was discussed.

## 2. Materials and Methods

### 2.1. Materials

CoNiCrAlY powder (15–45 um, AMDRY9951, SulzerMetco) and Al_2_O_3_ powder (50~200 nm, Al_2_O_3_ content ≥ 99.7%, Changsha Tianjiu Metal Material Co., Ltd., Changsha, China) were used as raw materials. 304 stainless steel was selected as the substrate. The chemical composition of CoNiCrAlY powder and 304 stainless steel were shown in Table 1 and Table 2, respectively.

### 2.2. Preparation of the Core-Shell Structured Powder

Before the experiment, the CoNiCrAlY powder and Al_2_O_3_ powder were dried at 100 °C for 2 h. Then, the CoNiCrAlY powder and the Al_2_O_3_ powder were mixed in a weight ratio of 10:1. Subsequently, CoNiCrAlY-Al_2_O_3_ core-shell structured powder was prepared with a DECO-PBM-2L planetary ball mill (Changsha Deke Instrument Equipment Co., Ltd., Changsha, China). The ball milling process parameters were as follows: the ball milling speed is 180 r/min, the ball-to-material weight ratio is 10:1, and the ball milling time is 6 h. In order to characterize the cross-sectional microstructure of the powder, the metallographic samples of the two powders were prepared in a XQ-1 metallographic sample mounting machine (Shanghai Caikang Optical Instrument Co., Ltd., Shanghai, China) using phenolic resin (Jinan Yuncheng Instrument Co., Ltd., Shanghai, China). Then, these mounted samples were ground with diverse sandpapers, and polished with 0.3 μm Al_2_O_3_ powder.

### 2.3. Preparation and Microstructure Characterization of HVOF Coatings

Before thermal spraying, the 304 stainless steel substrate is cleaned with acetone solution to remove grease. Then, sandblasting was performed on the surface of the 304 stainless steel substrate. The coating preparation was carried out on a JP-8000 HVOF system (Praxair, Inc., Praxair, America). The spraying process parameters were shown in Table 3.

The phases of core-shell structured powder and coatings were identified by AL-2700B X-ray diffraction analyzer (Dandong Oron Ray Instrument Co., Ltd., Dandong, China). The test parameters were as follows: the target material is Cu, the working voltage was 40 kV, the working current was 30 mA, and the scanning speed was 5°/min. The cross-sectional microstructure of CoNiCrAlY and CoNiCrAlY-Al_2_O_3_ coatings were characterized by Phenom proX scanning electron microscope (Funa Scientific Instruments Co., Ltd., Shanghai, China). The average porosity and oxide content of the coating are measured by the image analysis software using the gray method. In order to ensure the reliability of the measurement results, the average porosity and oxide content of the coatings are the average of the test results obtained from ten randomly selected SEM pictures. Similar measurement methods are consistent with those in the literature [26,27].

### 2.4. Electrochemical Measurements

Potentiodynamic polarization (PDP) and electrochemical impedance spectroscopy (EIS) were carried out to investigate the corrosion behavior of these coatings. The electrochemical station (CHI660E, Shanghai Chenhua Instrument Co., Ltd., Shanghai, China) with a three-electrode cell system was used. All the measurements were conducted in 3.5 wt.% NaCl solution at room temperature (~25 °C). The test samples were welded with a copper wire and cold-mounted in epoxy. An exposed surface area of 1 cm^2^ was served as a working surface. A platinum sheet and a saturated calomel electrode (SCE) were used as counter electrode and reference, respectively.

Before testing, the samples is monitored for open circuit potential (OCP) for about 30 min. Then, the EIS measurements were performed in a frequency range from 100 kHz down to 10 mHz at OCP using a 10 mV signal amplitude. The PDP tests were started from −0.5 V and ended at 1.5 V versus OCP at a potential scan rate of 1 mV/s. The surface morphology of the corroded sample after potentiodynamic polarization is characterized with a Phenom proX scanning electron microscope (Shanghai, China).

## 3. Results

### 3.1. Microstructure of the Core-Shell Structured Powder

**Figure 1:**
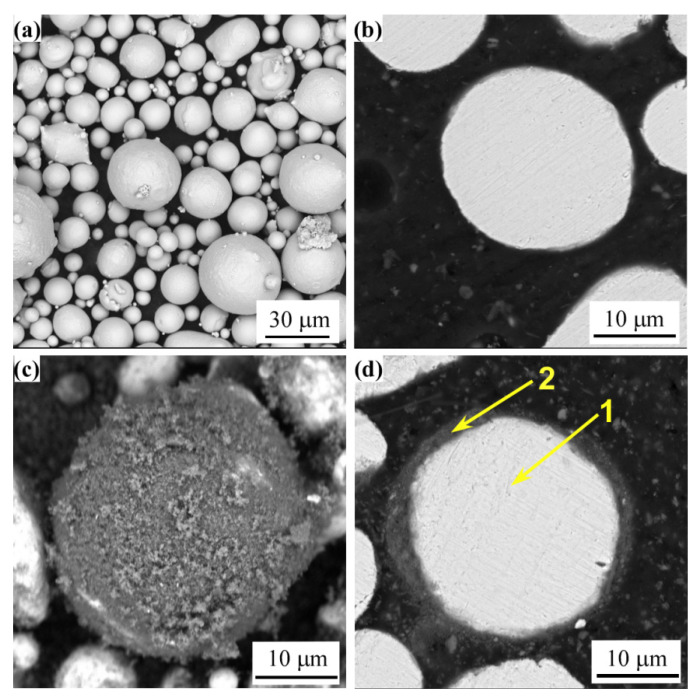
(**a**) Morphology of the CoNiCrAlY powder; (**b**) Cross-section morphology of the CoNiCrAlY powder; (**c**) Morphology of the CoNiCrAlY-Al_2_O_3_ core-shell structured powder; (**d**) Cross-section morphology of the CoNiCrAlY-Al_2_O_3_ core-shell structured powder.

Figure 1 shows the surface and cross-sectional morphologies of CoNiCrAlY raw powder and CoNiCrAlY-Al_2_O_3_ core-shell structured powder. The CoNiCrAlY powder has a smooth surface and a regular spherical shape. The microstructure of the CoNiCrAlY powder is uniform, and there are no defects such as pores. Figure 1c shows that the core-shell structured powder prepared by mechanical ball milling has an ellipsoidal shape, and the surface of the CoNiCrAlY powder is uniformly covered with a layer of gray Al_2_O_3_ particles. The outer layer of the Al_2_O_3_ shell adsorbs a small amount of loose Al_2_O_3_ powder. Figure 1d shows that the thickness of the Al_2_O_3_ shell of the core-shell structured powder is about 1 μm. The EDS analysis results in Table 4 prove that the gray area on the surface of the core-shell structured powder is an Al_2_O_3_ shell.

The key factor for preparing CoNiCrAlY-Al_2_O_3_ core-shell structured powders by mechanical ball milling is to choose the difference in particle size distribution of CoNiCrAlY and Al_2_O_3_ powders. During the ball milling process, through the continuous collision and extrusion between the powder and the steel ball, the Al_2_O_3_ particles with a smaller particle size will be squeezed into the surface of the CoNiCrAlY particles to form a core-shell structure. Therefore, in general, the larger the difference in particle size distribution and hardness between the core material and the shell material, the easier it is to obtain the core-shell structured powder [28,29]. Literature studies have shown that the ball milling process parameters have a great influence on the integrity of the core-shell structured powder [25,28]. In our previous work, we have optimized the mechanical ball milling process parameters through orthogonal experiments [25]. In addition, the surface coating integrity of the core-shell structured powder was evaluated by the gray scale method. Based on the optimized process parameters, the average coating rate of the core-shell structured powder used in this research can reach 69% [25].

### 3.2. Phase Structure of HVOF Sprayed Coatings

**Figure 2:**
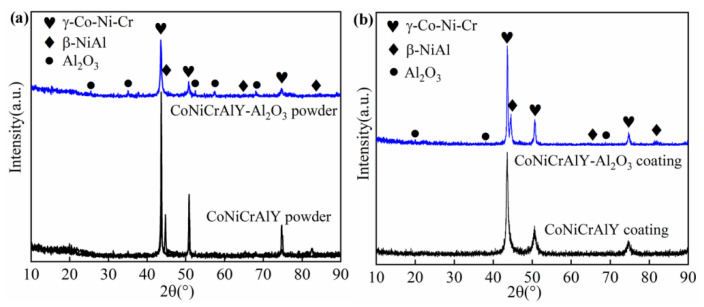
XRD patterns of powders (**a**) and these HVOF sprayed coatings (**b**) [25].

Figure 2 shows the XRD patterns of the prepared powders and HVOF sprayed coatings [25]. It can be seen that the original CoNiCrAlY powder was composed of γ (Co, Ni, Cr) solid solution and β-NiAl phase. This is consistent with the results reported in the literature [24]. Whereas, based on the core-shell structured powder design, the CoNiCrAlY-Al_2_O_3_ powder consists of γ (Co, Ni, Cr) solid solution, β-NiAl, and Al_2_O_3_.

As shown in Figure 2b, the CoNiCrAlY coating is mainly composed of γ (Co, Ni, Cr) phase, and there is almost no β phase. This may be due to the consumption of β-NiAl phase due to the oxidation of the powder during the spraying process [30]. In addition, the β-NiAl phase may be redissolved in the γ (Co, Ni, Cr) matrix in the molten CoNiCrAlY droplets. Due to the high cooling rate during the thermal spraying process, the β-NiAl phase does not have enough time to reprecipitate in the γ (Co, Ni, Cr) matrix [31].

However, the significant difference is that the CoNiCrAlY-Al_2_O_3_ composite coating prepared with the core-shell structured powder is composed of γ(Co, Ni, Cr) phase, Al_2_O_3_ phase, and β-NiAl phase. Except for Al_2_O_3_, the phase structure of the coating is highly consistent with that of the CoNiCrAlY powder. The relative intensity of the diffraction peaks of the β-NiAl phase did not decrease significantly, indicating that the Al_2_O_3_ shell significantly inhibited the oxidation behavior. In addition, the temperature changes of the droplets during the spraying process are also severely affected.

### 3.3. Microstructure of HVOF Sprayed Coatings

**Figure 3:**
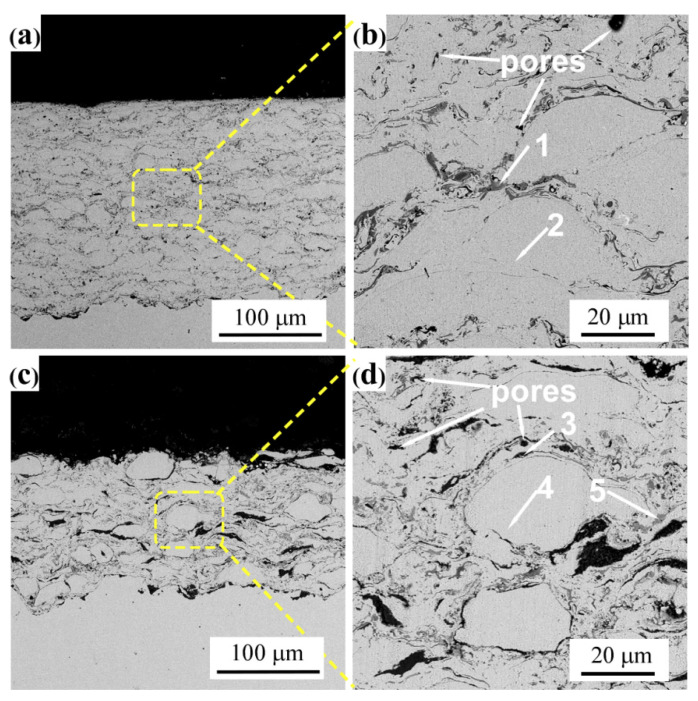
Cross-sectional morphologies of these of the coatings: (**a**,**b**) CoNiCrAlY coating; (**c**,**d**) CoNiCrAlY-Al_2_O_3_ coating.

Figure 3 illustrates the microstructure of HVOF CoNiCrAlY and CoNiCrAlY-Al_2_O_3_ coatings. It is apparent that the microstructure of the CoNiCrAlY coating is uniform and compact, with a typical layered microstructure. During the spraying process, the oxide layer on the surface of the droplets is distributed in the form of oxide strips in the coating. The average thickness of the CoNiCrAlY coating is 388 μm. The average porosity is about 1.6%, and the average oxide content is about 7.6%. There are few semi-melted particles and unmelted particles inside the coating. The bonding interface between the stainless steel substrate and the coating occludes each other, indicating a high bonding strength [32].

However, the CoNiCrAlY-Al_2_O_3_ coating prepared with the core-shell structured powder exhibits a completely different microstructure. The average thickness of the coating is significantly reduced to only 150 μm, indicating that the deposition rate of the powder during the spraying process is significantly reduced. The average porosity of the coating is significantly increased up to 3.9%. In addition, the number of unmelted particles in the coating has increased significantly. It can be concluded that the core-shell structured powder is not completely melted during the spraying process. This may be attributed to the high melting point of the Al_2_O_3_ shell and the high cooling rate of the molten droplets during the thermal spraying process (greater than 10^4^ °C/s). Therefore, the CoNiCrAlY droplets stays in the molten state for a relatively short time. As a result, the molten droplets possess a poor deformability during the collision with the surface of the substrate.

Table 5 shows the EDS analysis results of the positions as shown in Figure 3b,d. It can be found that the oxides in the CoNiCrAlY coating are concentrated at the edges of the deformed particles. This result is similar to that reported by Verdian et al. [33] and Silveira et al. [34]. In the CoNiCrAlY-Al_2_O_3_ coating, oxides are distributed at the interface of the layered structure and inside the deformed metal particles. EDS analysis results show that the oxide inside the CoNiCrAlY-Al_2_O_3_ coating contains a large amount of Al_2_O_3_, which is mainly derived from the Al_2_O_3_ shell in the core-shell structured powder. The metal particles inside the coating were also slightly oxidized. However, the average oxide content of the CoNiCrAlY-Al_2_O_3_ coating is only 5.1%, which is significantly less than that of the CoNiCrAlY coating. This indicates that the core-shell structured powder has a good inhibitory effect on the oxidation of CoNiCrAlY particles during the spraying process.

### 3.4. Corrosion Resistance of HVOF Coatings

#### 3.4.1. Potentiodynamic Polarization Curves

**Figure 4:**
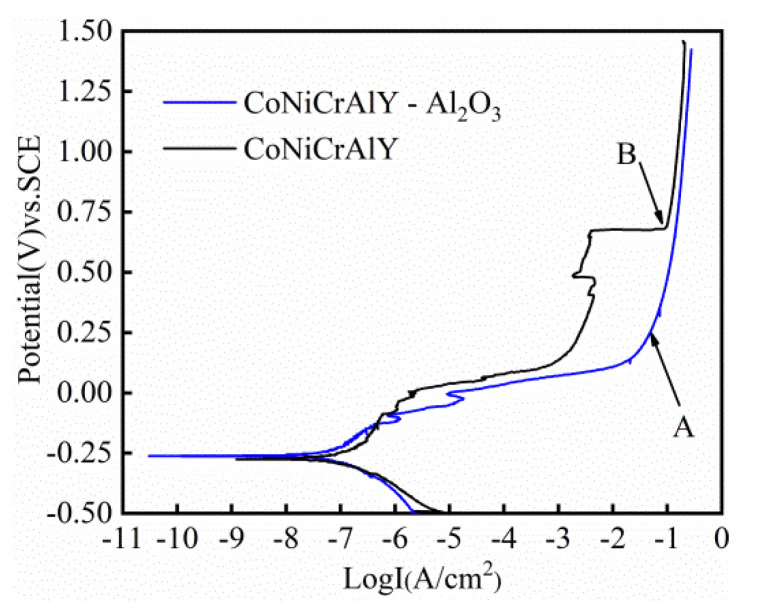
Potentiodynamic polarization curves of the HOVF sprayed CoNiCrAlY coating and CoNiCrAlY-Al_2_O_3_ coating.

Figure 4 displays the potentiodynamic polarization curves of the HVOF CoNiCrAlY and CoNiCrAlY-Al_2_O_3_ coatings in 3.5 wt.% NaCl solution at room temperature. As illustrated by the curves, both of the coatings display typical active-passive transitions. A series of electrochemical parameters, such as corrosion potential (*E_corr_*), corrosion current density (*I_corr_*) and passivation current density (*I_pass_*) fitted from PDP curves are listed in Table 6. The Zview program was used for calculations of anodic (*β_a_*) and cathodic (*β_c_*) Tafel constants required for polarization resistance calculations. The polarization resistance (*R_p_*) of coatings was calculated by using the Stern-Geary Equation (1) [35].

(1) $$R_{p}=\frac{dE}{di}=\frac{1}{i_{corr}}\frac{\beta_{a}\beta_{c}}{2.303(\beta_{a}+\beta_{c})}$$

where *i_corr_* is the corrosion current density. The *β_a_* and *β_c_* are the slope of anodic and cathodic Tafel lines respectively.

It can be found from Figure 4 that when the corrosion potentials are −108 and 405 mV, local passivation occurs on the surface of the CoNiCrAlY coating, but the passivation film is very unstable. When the corrosion potential reached 670 mV, the corrosion current density of the CoNiCrAlY coating increased sharply, and then a repassivation process occurred.

CoNiCrAlY-Al_2_O_3_ coating exhibits a high corrosion potential, low corrosion current density and large polarization resistance. It is inferred that the corrosion resistance of the coating is better than that of the CoNiCrAlY coating. The CoNiCrAlY-Al_2_O_3_ composite coating also has three stages of passivation during the entire polarization process. When the corrosion potentials are −108 and −26 mV, local passivation appeared on the coating surface. However, the passivation film is extremely unstable and is quickly re-dissolved. When the corrosion potential increases to 250 mV, a complete and dense passivation film form on the surface of the coating, and the corrosion current remained stable. It can be clearly found that the passivation potential of the coating is significantly lower than that of the CoNiCrAlY coating.

To better understand the corrosion property of the CoNiCrAlY-Al_2_O_3_ coating, we have also compared it with the CoCrNi medium entropy alloy and 316 L stainless steel (316L SS). Table 6 also provides the electrochemical parameters of the CoCrNi medium entropy alloy and 316L SS tested under similar experimental conditions [23,36]. Compared with the CoCrNi medium entropy alloy and 316L SS, the CoNiCrAlY-Al_2_O_3_ coating shows a lower corrosion current density and a higher corrosion potential, suggesting that the CoNiCrAlY-Al_2_O_3_ coating even has a better corrosion resistance than the CoCrNi medium entropy alloy and 316L SS.

#### 3.4.2. Electrochemical Impedance Spectroscopy

**Figure 5:**
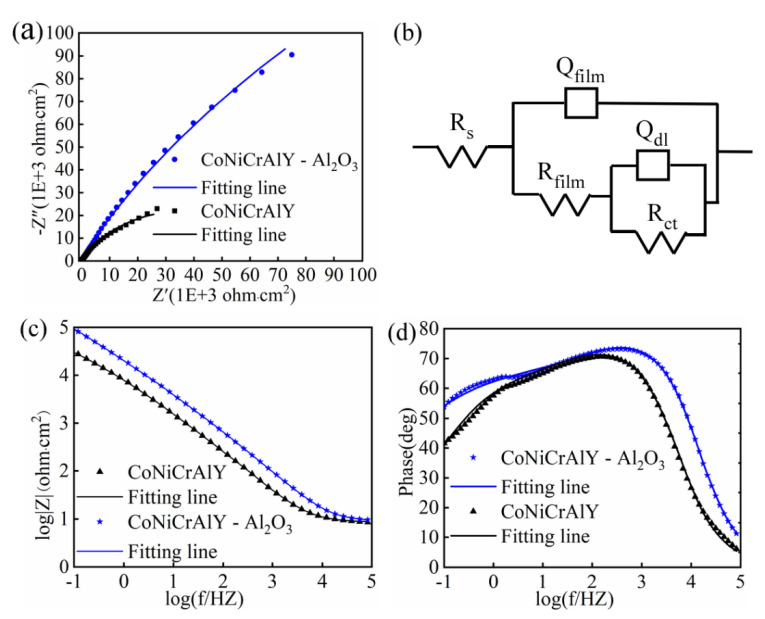
(**a**) Nyquist, (**b**) electrical equivalent circuit (EEC), and (**c**,**d**) Bode plots of the CoNiCrAlY-Al_2_O_3_ coating and CoNiCrAlY coating in 3.5 wt.% NaCl solution under OCP condition.

The EIS measurement results of the HOVF sprayed CoNiCrAlY coating and CoNiCrAlY-Al_2_O_3_ coating under OCP conditions are shown in Figure 5. The Nyquist plots of both of the coatings in the NaCl solution are incomplete semicircles (Figure 5a), which indicates that the corrosion process of the coating is controlled by charge transfer process. In general, the larger the capacitive arc diameter, the better the corrosion resistance of the coating [37]. It is obvious from Figure 5a that the capacitive arc diameter of the CoNiCrAlY-Al_2_O_3_ coating is significantly larger than that of the CoNiCrAlY coating, indicating that the coating has a higher resistant to electrochemical dissolution and a more protective passive film formed on its surface. In the Bode plots of Figure 5c, the slope of the *logZ-logf* curve of each sample is close to −1, which means that the passive film formed on the coating is pseudo-capacitive. In the Phase-logf diagram shown in Figure 5d, both coatings contain two peaks, which implies two relaxation time constant. The EIS of this study is simulated with Zsimpwin software by using the *R*_s_(*Q*_film_(*R*_film_(*Q*_dl_*R*_ct_) equivalent circuit proposed in references [37,38,39]. Among them, the *R*_s_, *R*_film_, and *R*_ct_ are the solution and charge-transfer as well as film resistances, respectively. The *Q*_film_ and *Q*_dl_ represent the equivalent capacitance of the passivation film and the electric double layer, respectively. These fitted parameters are presented in Table 7. The equivalent circuit diagram is shown in Figure 5b. The Chi-squared (χ^2^) value in Table 7 can be used to evaluate the fitting accuracy of the data [40]. The Chi-squared (χ^2^) values of the two coatings are in the order of 10^−4^, indicating a high fitting accuracy.

Since the passivation film on the surface of these coatings is not uniform, their capacitance is represented by the equivalent capacitance Q (constant phase angle element CPE) [41]. The Q includes two parameters: constant Y_0_ (Ω^−1^·cm^−2^·s^–n^) and dimensionless index *n*. The *n* is the value for the deviation from pure capacitance behavior. The closer the value of *n* is to 1, the more the system behaves as an ideal capacitance [42]. According to the literature [43,44], the capacitance is closely related to the thickness and compactness of the passivation film. According to the data in Table 7, it is obvious that the *Q*_film_-*Y*_0_ value (4.9 × 10^−6^ Ω^−1^·cm^−2^·s^–n^) of the CoNiCrAlY-Al_2_O_3_ coating is much lower than that of the CoNiCrAlY coating, indicating that the passivation film on the surface of the coating is thicker and denser. In addition, the high *R*_s_ (9.1 Ohm·cm^2^) value of the CoNiCrAlY-Al_2_O_3_ coating indicates that the electrochemical reaction rate that occurs on the surface of the coating is slow. The high *R*_film_ and *R*_ct_ values of the CoNiCrAlY-Al_2_O_3_ coating indicate that the passivation film on the coating surface is stable and the sensitivity to anions in the solution is low.

#### 3.4.3. Surface Morphologies of Corroded Coatings

**Figure 6:**
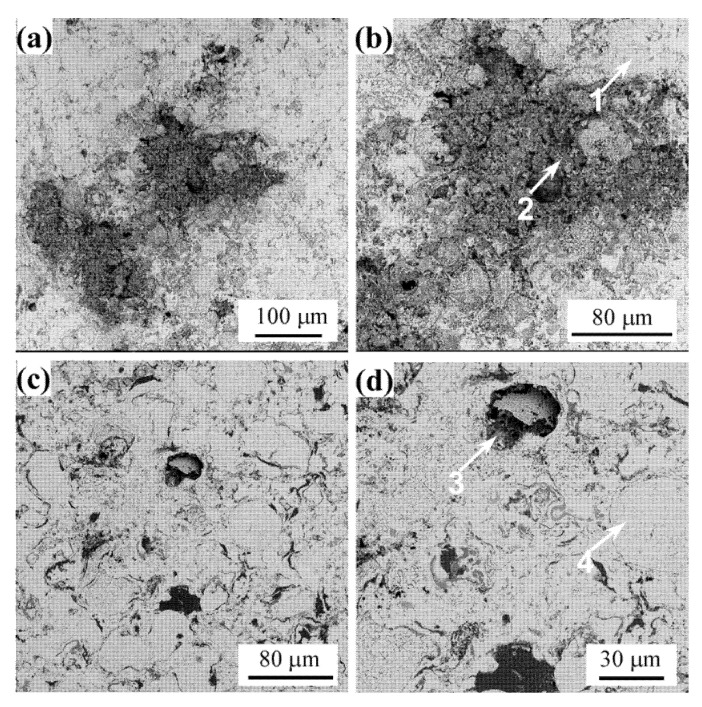
Surface morphologies of these corroded coatings: (**a**,**b**) CoNiCrAlY coating; (**c**,**d**) CoNiCrAlY-Al_2_O_3_ coating.

In order to further clarify the corrosion mechanism of the two coatings in 3.5 wt.% NaCl solution, SEM and EDS analysis was performed on the surface of these coatings after the potentiodynamic polarization test. Figure 6 shows the surface morphologies of the corroded CoNiCrAlY coating and CoNiCrAlY-Al_2_O_3_ coating. It is obvious from Figure 6 that the preferentially corroded areas on the surface of the two coatings are at the boundary of the incompletely melted spherical particles. Figure 6a shows that there is a large area of interconnected corrosion zone on the surface of the CoNiCrAlY coating. However, in Figure 6c, the corrosion occurred on the surface of the CoNiCrAlY-Al_2_O_3_ coating is slight. EDS analysis was performed on the marked locations in Figure 6b,d, and the results are shown in Table 8. The corrosion products on the surface of the CoNiCrAlY coating consist of Ni/Co/Cr oxides and chlorides. However, the corrosion products formed on the surface of the CoNiCrAlY-Al_2_O_3_ coating are mainly Al_2_O_3_, which may be related to the Al_2_O_3_ shell of the core-shell structured powder.

## 4. Discussion

### 4.1. Influence of Core-Shell Structured Powder on the Phase Structure and Microstructure of HVOF Coatings

The above results indicate that the core-shell structured powder has a significant effect on the phase composition and microstructure of the HVOF coatings. It can be seen from Figure 2 that there is almost no β-NiAl phase in the CoNiCrAlY coating. The reason is mainly due to the oxidation of the powder during the spraying process. In addition, when the powder is in a high-temperature molten state, the original β-NiAl phase in the CoNiCrAlY alloy may be dissolved in the γ-(Co, Ni, Cr) solid solution again [26]. However, the XRD pattern of the CoNiCrAlY-Al_2_O_3_ composite coating showed a strong β-NiAl phase diffraction peak. Except for the external Al_2_O_3_ shell material, the phase structure of the CoNiCrAlY-Al_2_O_3_ composite coating and the CoNiCrAlY powder are almost the same. This is mainly due to the following two reasons.

First, the Al_2_O_3_ shell of the core-shell structured powder obviously prevents the oxidation of the CoNiCrAlY powder during the spraying process. The β-NiAl phase in CoNiCrAlY powder has a high affinity for oxygen and is prone to selective oxidation. However, during the spraying process, the nano Al_2_O_3_ shell isolates the molten CoNiCrAlY core powder from oxygen very well. Huang et al. [45] and Tian et al. [18] prepared thermal spray coatings with (Cr_3_C_2_-BaF_2_-CaF_2_)-NiCr and NiCr-Mo core-shell structured powders, respectively. Similar phenomena have been reported.

Second, the core-shell structured powder significantly changes the temperature field of the droplet particles during the spraying process. Tian et al. [18] prepared a core-shell structured powder with NiCr metal powder, which was coated with a molybdenum shell. The melting point of molybdenum is 2620 °C, and its specific heat and thermal conductivity at room temperature are 242.8 J/(kg·°C) and 142.3 W/(m·°C), respectively [46]. The combination of high thermal conductivity and low heat capacity enables molybdenum to heat up and cool quickly. According to the author’s report, during the thermal spraying process, the molybdenum shell extends the time that the NiCr core powder staying in the molten state. This leads to enhanced deformability of the droplet particles during the collision with the substrate, which improves the compactness of the coating [18]. However, molybdenum is easily oxidized in a medium temperature environment below 1000 °C [18]. In this study, α-Al_2_O_3_ was selected as the outer shell material of the core-shell structured powder. The melting point of α-Al_2_O_3_ is 2054 °C, and its specific heat and thermal conductivity at room temperature are 750 J/(kg·°C) and 10 W/(m·°C), respectively [47]. Compared with molybdenum, the α-Al_2_O_3_ shell material has high specific heat capacity and low thermal conductivity. This will lead to a decrease in the temperature of the CoNiCrAlY core material during the thermal spraying process. The time that the core-shell structured powder staying in the molten state is shortened. As a result, the deformability of the droplets is poor, resulting in a high porosity and high content of unmelted particles in the coating. In addition, the deposition rate of the powder during the thermal spraying process decreases. This is consistent with the influence of the core-shell structured powder on the evolution of the coating microstructure shown in Figure 3.

In addition, another reason for the higher porosity of the CoNiCrAlY-Al_2_O_3_ composite coating is the large difference in thermal expansion coefficient between the CoNiCrAlY alloy and the Al_2_O_3_ shell. Tian et al. [18] also reported similar results. Wang et al. [48] prepared the coating with Ni60-TiO_2_ core-shell structured powder as the raw material. Studies have shown that the pores are distributed near the unmelted nano-TiO_2_ particles in the coating. The porosity and the number of unmelted particles in the coating increase.

### 4.2. The Effect of Core-Shell Structured Powder on the Corrosion Behavior of HVOF Coatings

**Figure 7:**
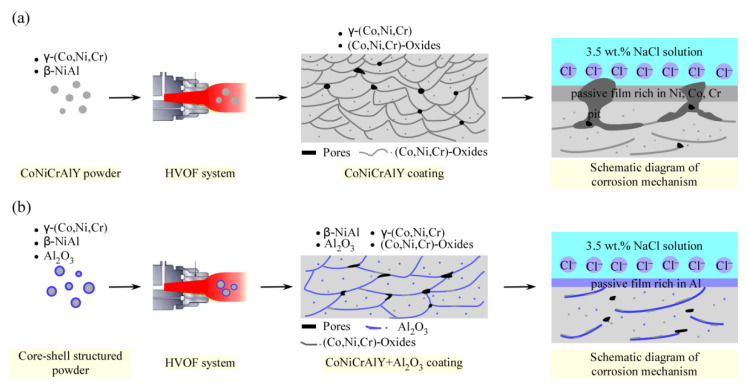
Schematic diagram of microstructure evolution and corrosion mechanism of the CoNiCrAlY coating (**a**) and CoNiCrAlY-Al_2_O_3_ coating (**b**).

As shown in Figure 5, the corrosion resistance of CoNiCrAlY-Al_2_O_3_ coating in 3.5 wt.% NaCl solution is significantly better than that of CoNiCrAlY coating. Figure 6 shows that a large area of pitting corrosion appears on the surface of the CoNiCrAlY coating. While the corrosion on the surface of the CoNiCrAlY-Al_2_O_3_ coating is obviously suppressed, and the passivation film on the surface is denser. It can be concluded that the interface of the layered structure and the interface of the unmelted particles are the preferential channels for corrosion. Figure 7 provides a schematic diagram of the microstructure evolution of CoNiCrAlY and CoNiCrAlY-Al_2_O_3_ coatings and their corrosion mechanism in 3.5 wt.% NaCl solution.

As shown in Figure 7a, the CoNiCrAlY coating contains almost no β-NiAl phase. The oxidation of the powder during the spraying process causes a large amount of Ni/Co/Cr/Al oxides in the coating. According to literature reports [49], during the thermal spraying process, Cr and Al in molten particles may be rapidly oxidized (because Cr and Al are highly sensitive to oxidation). This results in the lack of sufficient Cr and Al in the coating and the poor stability of the passivation film formed on the surface. The oxide layer in the coating is not dense, causing the coating’s corrosion resistance to be worse than that of the alloy with the same chemical composition [33,50]. In the 3.5 wt.% NaCl solution, the Ni/Co/Cr oxide in the CoNiCrAlY coating will be corroded and dissolved, and a new passivation film will be formed on the surface of the coating. Micro-galvanic corrosion is prone to occur between oxides and metals, thereby accelerating the corrosion rate of the coating. Defects such as pores and oxide interfaces in the coating help chloride ions to penetrate the coating [34,51]. These phenomena lead to pitting corrosion on the surface of the coating and destroy the continuity of the passivation film. Hao et al. [37] showed that the passivation film on the surface of the CoNiCrAlYTa coating is also not continuous. Studies have shown that the accumulation of Ni^2+^ in the corrosion pits due to the dissolution of the coating is conducive to the diffusion of Cl^−^ to maintain the balance of charges [52]. The diffusion of ions will change the electrolyte composition and chemical properties in the pores, leading to local acidification and further corrosion [53]. Literature studies have shown that [54] the passivation film on the surface of the CoNiCrAlY coating is mainly composed of oxides and hydroxides of Ni, Co, and Cr elements.

Compared with the CoNiCrAlY coating, the content of β phase and Al_2_O_3_ in CoNiCrAlY-Al_2_O_3_ coating is higher. It is reported that the β-NiAl phase is an important source of Al element for the formation of Al-rich surface passivation film of CoNiCrAlY alloy in corrosive environment [55]. Therefore, a higher β-NiAl phase content is beneficial to improve the corrosion resistance of the coating. Furthermore, due to the core-shell structured powder, the oxidation of the CoNiCrAlY core alloy has been significantly inhibited. The consumption of Co/Ni/Cr/Al in the CoNiCrAlY core alloy due to oxidation during the spraying process is significantly reduced. Therefore, the repassivation ability of the CoNiCrAlY-Al_2_O_3_ coating in a corrosive environment can be significantly improved. In addition, due to the poor deformability of the core-shell structured droplets, the thickness of the layered structure inside the CoNiCrAlY-Al_2_O_3_ coating becomes larger, the interface between the layered structures is reduced. Then, the preferential channel for corrosion is reduced. Because of this, the Al_2_O_3_ shell material is distributed at the interface of the layered structure in the as-sprayed coating, a large number of nucleation sites for the Al_2_O_3_-riched passivation film are provided, which helps to improve the continuity and compactness of the passivation film. For the above reasons, in the 3.5 wt.% NaCl solution, the passivation film formed on the surface of the CoNiCrAlY-Al_2_O_3_ coating is rich in Al and denser, as shown in Figure 7b. Even if the porosity in the CoNiCrAlY-Al_2_O_3_ coating increases, due to the higher passivation ability of the coating and the high density and continuity of the passivation film, the corrosion resistance of the coating does not decrease significantly. Jiang et al. [16] prepared a plasma spray coating with Ni/Al coated iron-based amorphous powder. The corrosion resistance of the coating has been significantly improved.

## 5. Conclusions

(1) The CoNiCrAlY-Al_2_O_3_ core-shell structured powder with Al_2_O_3_ as the shell was successfully prepared by mechanical ball milling. The thickness of the Al_2_O_3_ shell of the core-shell structured powder is about 1 μm.
(2) The temperature field of the molten CoNiCrAlY powder during the HVOF spraying process is significantly changed by the Al_2_O_3_ shell. The poor deformability of the CoNiCrAlY-Al_2_O_3_ droplets leads to an increase in the porosity and unmelted particles of the coating. In addition, the significant difference is that the CoNiCrAlY-Al_2_O_3_ coating also maintains a high content of β-NiAl phase. The lower oxide content in the coating indicates that the core-shell structured powder significantly inhibits the oxidation of the CoNiCrAlY core powder during the spraying process.
(3) The HVOF sprayed CoNiCrAlY-Al_2_O_3_ coating exhibits excellent corrosion resistance in 3.5 wt.% NaCl solution. The formation of a continuous dense Al-rich passivation film on the coating is mainly due to the high content of Al_2_O_3_ and β-NiAl phase.

## Figures and Tables

**Table 1:** Chemical composition of CoNiCrAlY (wt.%) [25].

Co	Ni	Cr	Al	Y
38.5	32	21	8	0.5

**Table 2:** Chemical composition of 304 stainless steel (wt.%) [25].

Fe	C	Mn	P	S	Si	Cr	Ni
Bal	0.08	2	0.045	0.03	1	18~20	8~11

**Table 3:** Parameters of HVOF spraying.

Spray Parameter	Value
Oxygen flow (SCPH)	2000
Carrier Gas flow (SCFH)	21
Kerosene flow (GPH)	5.5
Spray distance (mm)	360
Gun moving Speed (mm/s)	300

**Table 4:** EDS analysis results of the positions as marked in Figure 1d (at.%).

Number	Co	Ni	Cr	Al	Y	O
1	38.99	28.85	19.30	7.64	0.14	5.08
2	-	-	-	46.55	-	53.45

**Table 5:** EDS analysis results of these positions as marked in Figure 3 (at.%).

Number	Co	Ni	Cr	Al	Y	O
1	26.66	12.30	17.67	10.12	0.97	32.27
2	38.99	28.55	19.30	7.64	0.14	53.45
3	-	-	-	46.55	-	53.45
4	33.64	30.79	17.83	11.33	0.67	5.74
5	23.86	13.77	17.22	15.95	0.42	28.77

**Table 6:** Electrochemical parameters obtained from the potentiodynamic polarization curve of these coatings.

	*E_corr_* (mV)	*I_corr_* (μA·cm^−2^)	*E_pass_* (mV)	*I_pass_* (A·cm^−2^)	*β_a_* (V·dec^−1^)	*β_c_* (V·dec^−1^)	*R_p_* (KΩ·cm^2^)
CoNiCrAlY-Al_2_O_3_	−251	0.066	321	0.065	0.15	−0.12	43.9
CoNiCrAlY	−269	0.17	691	0.096	0.34	−0.15	20.2
CoNiCr [36]	−320	1.4	-	-	-	-	-
316L SS [23]	−273	0.213	-	-	-	-	-

**Table 7:** Electrochemical parameters obtained from electrochemical impedance spectroscopy of coatings.

	*R*_s_ (Ohm·cm^2^)	*Q*_film_-*Y*_0_ (Ω^−1^·cm^−2^·s^−n^)	*Q*_film_-*n*	*Q*_dl_-*Y*_0_ (Ω^−1^·cm^−2^·s^−n^)	*Q*_dl_-*n*	*R*_film_ (Ohm·cm^2^)	*R*_ct_ (Ohm·cm^2^)	χ^2^ (×10^−4^)
CoNiCrAlY-Al_2_O_3_	9.1	4.9 × 10^−6^	0.88	9.4 × 10^−6^	0.58	2839	797,300	1.98
CoNiCrAlY	8.6	1.5 × 10^−5^	0.85	2.2 × 10^−5^	0.56	2388	77,250	6.16

**Table 8:** EDS analysis results of the positions as marked in Figure 6 (at.%).

Number	Co	Ni	Cr	Al	Y	O	Cl
1	36.94	29.11	20.13	7.47	0.35	5.99	-
2	23.98	20.60	16.60	4.97	1.08	18.32	14.99
3	5.25	4.61	3.11	43.04	0.59	42.41	0.99
4	36.31	29.75	19.21	8.30	0.51	6.10	-

## Data Availability

Data sharing not applicable.
